# rTMS targeted to the secondary somatosensory cortex influences sleep in CRPS patients, as measured with the OURA ring

**DOI:** 10.1002/brb3.3252

**Published:** 2023-09-12

**Authors:** Jukka Vanhanen, Jan Kujala, Mia Liljeström, Eija Kalso, Jussi Virkkala, Hanna Harno

**Affiliations:** ^1^ HUS Diagnostic Center, Clinical Neurophysiology, Clinical Neurosciences Helsinki University Hospital and University of Helsinki Helsinki Finland; ^2^ BioMag Laboratory, HUS Diagnostic Center Helsinki University Hospital and University of Helsinki Helsinki Finland; ^3^ Department of Psychology University of Jyväskylä Jyväskylä Finland; ^4^ Department of Neuroscience and Biomedical Engineering Aalto University Espoo Finland; ^5^ Department of Anaesthesiology, Intensive Care and Pain Medicine Helsinki University Hospital and University of Helsinki Helsinki Finland; ^6^ SleepWell Research Program University of Helsinki Helsinki Finland; ^7^ Clinical Neurosciences, Neurology Helsinki University Hospital and University of Helsinki Helsinki Finland

**Keywords:** CRPS, rTMS, secondary somatosensory cortex, sleep

## Abstract

**Introduction:**

Chronic pain associates with various sleep problems. Patients with complex regional pain syndrome (CRPS) often report impaired sleep, but objective measurements of sleep in CRPS patients are scarce. Neuromodulation with repetitive transcranial magnetic stimulation (rTMS) can alleviate pain and improve sleep. Secondary somatosensory cortex (S2) is a possible rTMS target for the treatment of chronic pain, but the effect of S2‐targeted rTMS on sleep is unknown.

**Methods:**

This randomized, sham‐controlled trial assessed the effect of S2‐targeted rTMS on sleep in patients with CRPS. Patients (*n* = 31) received either S2‐targeted rTMS (10 Hz) or sham stimulation for 3 weeks. The effect of treatment on sleep was assessed with validated questionnaires, with a sleep and pain diary, and with a consumer‐grade sleep tracker, the Oura ring. In addition to an ordinary univariate analysis of the results, we conducted multivariate testing of the Oura data using linear discriminant analysis (LDA).

**Results:**

S2‐targeted rTMS decreased sleep restlessness that significantly differed between the rTMS and sham stimulation patient groups (*p* = .028). In the multivariate analysis of the Oura data, LDA classification accuracy to separate the rTMS and sham groups exceeded 95% confidence level in four out of the seven tested models. In the subjective evaluation of sleep, the effect of rTMS and sham did not differ.

**Conclusion:**

S2‐targeted rTMS influenced sleep in patients with CRPS. Improved sleep may enhance CRPS symptom alleviation and be of clinical importance. A univariate analysis could separate the rTMS and sham treatments. The multivariate analysis revealed that including multiple sleep‐related parameters can be beneficial when analyzing rTMS effects on sleep. As sleep is related both to pain and quality of life, and sleep rTMS can be directly affected by rTMS, objective monitoring of sleep in various future rTMS trials could be fruitful.

## Graphical abstract

Repetitive transcranial magnetic stimulation (rTMS) targeted at the secondary somatosensory cortex decreased sleep restlessness in patients with complex regional pain syndrome. A multivariate analysis of OURA parameters revealed that assessing multiple sleep‐related parameters concurrently can be beneficial when analyzing rTMS effects on sleep.

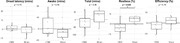

## 1 INTRODUCTION

Insufficient sleep and pain are highly interrelated, and sleep disturbance is a risk factor for developing and exacerbating chronic pain both in the general population and in those with various pain conditions (Finan et al., 2013; Simpson et al., 2018). For example, most patients with fibromyalgia or rheumatoid arthritis report poor sleep quality (Bigatti et al., 2008; Sariyildiz et al., 2014). Manifestations of fragmented sleep, such as increased wakefulness after sleep onset (WASO), increased number of awakenings, reduction of total sleep time (TST), prolonged sleep onset latency (SOL), and decreased sleep efficiency (SE), are common findings in the polysomnography (PSG) of chronic pain patients (Bjurstrom & Irwin, 2016). Relatively little is known, however, about the role of sleep in complex regional pain syndrome (CRPS). To the best of our knowledge, only one study has assessed sleep in CRPS patients with objective measurements, revealing a high number of awakenings and arousals in all five examined patients (van de Beek et al., 2002).

Repetitive transcranial magnetic stimulation (rTMS) is a noninvasive neuromodulation method that has proven efficacious in neuropathic pain. A few studies have reported analgesic effects of rTMS also in CRPS patients (Picarelli et al., 2010; Pleger et al., 2004). For the analgesic effect, rTMS is usually targeted to the primary motor cortex. We have recently shown that the secondary somatosensory cortex (S2) may also be a promising target for rTMS (Ojala et al., 2021). rTMS also appears effective in certain sleep disorders, especially primary insomnia (PI) and restless legs syndrome (RLS), whereas rTMS results in many other sleep disorders are inconsistent (Lanza et al., 2023; Oroz et al., 2021). Importantly, studies assessing the efficacy of rTMS on sleep with objective measurements, for example, PSG, are scarce. In patients with insomnia, rTMS targeted to the frontal cortex resulted in improvements of PSG parameters TST, SE, SOL, and WASO (Sánchez‐Escandón et al., 2014), and rTMS targeted to the right dorsolateral prefrontal cortex improved sleep architecture (Jiang et al., 2013).

The exact mechanisms of how noninvasive brain stimulation improves sleep are only partially known. Some sleep disorders associate with distinctive patterns of neurophysiological alterations, which provide a rationale for different neuromodulatory protocols (Lanza et al., 2023). The local effects of specific rTMS protocols are reasonably well studied, but wide‐ranging network effects, for example, via neurotransmitter release, remain poorly understood (Dayan et al., 2013; Fitzgerald et al., 2006). One proposed mechanism for neuromodulative sleep enhancement is the restoration of sleep homeostasis and neural plasticity (Lanza et al., 2022; Lanza et al., 2023). Data about the effect of S2‐targeted rTMS on sleep is limited. In patients with neuropathic orofacial pain, a single session of S2‐targeted rTMS did not affect self‐reported sleep quality (Lindholm et al., 2016). No studies have so far assessed the effect of S2‐targeted rTMS on sleep with objective measurements.

This study explores sleep in CRPS patients by assessing polygraphic biosignals recorded by a multisensory sleep tracker, the Oura ring. Several validation studies have compared the Oura ring with PSG (De Zambotti et al., 2019; Kinnunen, 2016; Miller et al., 2022; Roberts et al., 2020), and Oura appears to detect sleep and wake accurately. We hypothesized that S2‐targeted rTMS has an effect on sleep, either independently or associated with possible analgesic effects, and aimed to assess whether we could use Oura ring to capture these changes. We examined different sleep parameters with a univariate analysis and, to gain a more comprehensive view of possible rTMS effects on sleep, also applied a multivariate analysis to the Oura data using a machine learning approach.

## 2 METHODS

This trial was part of a multicenter‐randomized controlled trial assessing the effect of rTMS targeted to the right S2 on pain in patients with CRPS. A single center (Helsinki) of this larger study included the Oura ring assessment of sleep in the study protocol. Results of the whole multicenter study, focusing on pain and quality of life, will be published elsewhere (manuscript in preparation).

### 2.1 Patients

**FIGURE 1:**
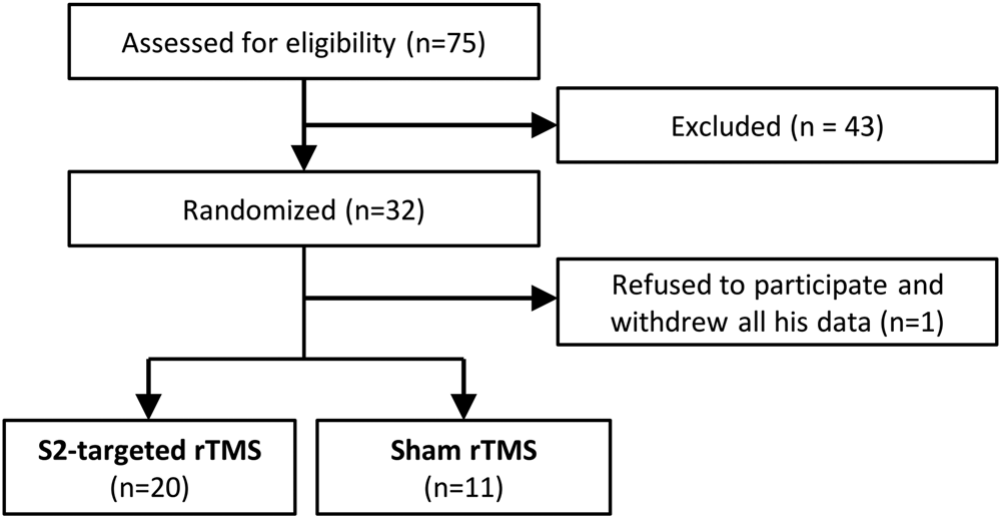
Flow diagram of patient enrollment.

Eligible patients who fulfilled the inclusion criteria (CRPS 1 or 2 of the upper limb, duration ≥6 months, age ≥18 years, mean pain intensity ≥5/10, conventional therapies have been tried or continue without significant relief) were recruited from the Helsinki University Hospital Pain Clinic, Private clinics, and via advertisements to social media in a private CRPS patient group (Figure 1). Exclusion criteria were other ongoing stimulation therapy apart from transcutaneous nerve stimulation, major psychiatric condition, neurodegenerative or other neurological disease, use of strong opioids, epilepsy, any contraindications to MRI or TMS, abuse of alcohol or drugs, and ongoing insurance or other entitlement cases.

The study was approved by the Helsinki University Hospital Ethics Committee and was registered in the ClinicalTrials.gov (NCT04439669). All eligible patients signed the informed consent.

### 2.2 Intervention

The patients were randomized into two parallel groups, rTMS and sham, in a 2:1 proportion. Each patient received 10 sessions of rTMS (biphasic figure of 8 coil, 10 Hz, trains of 100 pulses, 30‐s inter train interval with a 10‐min break in the middle of each session, summing up to 1500 pulses per session) during a 3‐week period (protocol adapted from a previous rTMS study targeting S2 (Lindholm et al., 2015)). The rTMS was targeted to the right S2 (irrespective of CRPS side) with an MRI‐guided navigation system (eXimia magnetic stimulator, Nexstim Plc), and the target was manually tagged on individual brain MRIs, based on known anatomic landmarks. The stimulation intensity for S2 was 90% of the individual resting motor threshold. For sham, we used a similar protocol as for the rTMS treatment, but we attached a 75‐mm plastic block between the TMS coil and scalp to attenuate the magnetic field reaching the brain. The patients, the screening neurologist, and the research nurse were blinded to the type of stimulation, whereas the researcher operating the TMS was aware of the stimulation type.

### 2.3 Sleep recordings with OURA ring

We used a multisensory sleep‐tracker, the Oura ring (Generation 1, Ōura Health), to assess sleep and other user‐derived biosignals, such as activity, heart rate variability, and skin temperature in the patients. Previous validation studies (De Zambotti et al., 2019; Kinnunen, 2016; Roberts et al., 2020) report that the Oura ring gives a good estimation of the following night‐level summaries of sleep, which we chose as the most reliable Oura‐variables to our study: TST (Oura variable “total”; total amount of sleep registered during the sleep period), SOL (“onset latency”; latency from the start of the sleep period to the beginning of the first 5 min of persistent sleep) and awake time after sleep onset (“awake”; total amount of awake time registered during the sleep period). As chronic pain can also associate with increased arousals during sleep (Abdulaziez & Asaad, 2012; Jennum et al., 1993; Mahowald et al., 1989; van de Beek et al., 2002), we also examined the parameter “restless” (percentage of sleep time when the user was moving) of the Oura data.

Patients were instructed to wear the Oura ring in the healthy hand for five consecutive days in two separate periods: The week before rTMS‐stimulation and the week after the stimulation had ended. Patients chose the best fitting Oura ring from different test ring sizes, and the research nurse collected the Oura data after recording periods. Patients were not allowed to access their Oura data during the trial.

### 2.4 Questionnaires

Patients filled in a set of validated questionnaires, including the Insomnia Severity Index (ISI) (Bastien et al., 2001) and Brief Pain Inventory (Cleeland & Ryan, 1994), before and 1 month after the intervention. In addition, the patients recorded daily their current pain intensity on a numeric rating scale (NRS) from 0 to 10 (0: no pain, 10: worst imaginable pain). Each morning the patients estimated how much the pain had interfered with their sleep. Patients kept this pain–sleep diary for 7 weeks, beginning 2 weeks before the rTMS intervention, during the 3‐week rTMS intervention, and for 2 weeks after it.

### 2.5 Statistics

Normally distributed questionnaire data, self‐reported pain, and sleep interference NRS values were analyzed with a mixed model analysis of variance (ANOVA) using a between‐subjects factor “treatment” and a within‐subject factor “time.” Correlation of different self‐reported parameters was tested with Pearson's product‐moment correlation.

In the evaluation of Oura data at baseline and after the intervention, we only included those recorded periods that comprised more than one night of Oura data in each patient. Across the patients, Oura data were collected during 5.1 ± 0.9 (mean ± SD) and 4.9 ± 1.2 nights in the pretreatment phase and during 4.4 ± 0.9 and 4.1 ± 1.3 nights in the posttreatment phase from the rTMS and sham groups. There were no differences in the number of nights between the two groups (independent samples *t*‐test, *p* > .65 both for the pre‐ and posttreatment phase).

We tested, with univariate statistical tests and in a multivariate machine learning framework, whether the rTMS‐treatment had induced changes in the sleep patterns between the pre‐ and posttreatment phases. Univariate testing was conducted by comparing the effect of rTMS and sham stimulation on the four Oura variables (change between the pre‐ and posttreatment phase in the median values across nights) using nonparametric Mann–Whitney *U*‐tests. Multivariate testing was conducted using linear discriminant analysis (LDA) to determine whether the two trial groups (sham and rTMS) could be separated from each other. We used the functions implemented in the MATLAB Statistics and Machine Learning Toolbox, and the LDA was conducted using the default parameters within the *fitcdiscr* function. In the LDA, we first tested whether the four variables showed significant correlations between each other by comparing the change in their mean values between the pre‐ and posttreatment phases across all variable combinations.

Significant linear correlation was detected between “awake” and “restless” (*p* = .027) and the correlation approached significance between “awake” and “onset_latency” and “total” (*p* = .070 and *p* = .072, respectively); no significant correlations were detected among “restless,” “onset_latency,” and “total” (*p* > .3 for all combinations). Accordingly, the variable “awake” was excluded from the multivariate analysis. In the LDA, we constructed seven different models representing all possible combinations of the three variables (“onset_latency,” “total,” and “restless”). That is, one model consisted of all three variables, whereas three different models were constructed with two variables and one variable, respectively.

In the LDA, we first trained the model across all patients using the differences between individual pre‐ and posttreatment nights in the Oura variables. Subsequently, the model was tested using Oura data from different individual nights than those used for the training data to determine how many patients the model could correctly classify to the right group. These phases were repeated 10,000 times by randomly sampling the individual nights used for the training and testing data. For each round, the mean classification accuracy across the 17 patients was stored, and the mean classification value across the 10,000 randomizations was taken as the total classification accuracy for each of the 7 models. The significance of the classification accuracy was evaluated by using permutation testing. This procedure followed the computation of the classification accuracy, but before the 10,000 random samplings of individual nights for the training and testing data, the labels of the rTMS and sham groups were permuted 1000 times. Here, we kept the sizes of the two groups the same as for the original data (10 patients in the rTMS and 7 in the sham group). The mean classification scores were stored for each permutation round, and the 95% confidence levels were computed from these distributions for each of the seven models. The *p*‐values of each model were computed by comparing the classification accuracy to this permutation‐based distribution.

Finally, to evaluate association of changes in pain with sleep parameters, we tested the correlation of change in each OURA parameter with the percentage of pain reduction (calculated as (NRS_POST_ − NRS_PRE_)/NRS_PRE_ × 100%) with Pearson's product‐moment correlation. Statistical analysis (except for multivariate testing) was conducted in R (R Core Team, 2020), and alpha level of .05 denoted statistical significance.

## 3 RESULTS

Of the 31 recruited patients, 21 had Oura data available from the pretreatment period of more than 1 night. Of these 21 patients, 17 patients had a complete data set with pre‐ and posttreatment Oura‐data and ISI questionnaires. The reported baseline values included all 21 patients, and the analysis of treatment effects included 17 patients (10 in rTMS group and 7 in sham group).

### 3.1 Self‐reported sleep and pain

At baseline, patients reported subthreshold (ISI score 8–14) to moderate (15–21) insomnia in the ISI questionnaire (Table 1). Pain interference with sleep (median of the week before the intervention) correlated positively with the ISI questionnaire (*r*(18) = .54, *p* = .013). There was a trend for sleep interference to associate with higher pain levels, but this correlation was not statistically significant (*r*(18) = .40, *p* = .083).

**TABLE 1:** Patient characteristics and questionnaire data at baseline.

Number of all patients	*n* = 21
Age (years)	46 (12)
Sex (female/male)	18 (86%)/3 (14%)
Duration of CRPS (years)	2.2 (0.7–16)
*>1 year*	17 (81%)
CRPS type (Type I/Type II)	15 (71%)/6 (29%)
BPI sleep interference	6.4 (2.7)
ISI score	13 (5.9)
Pain–sleep diary (pre‐week median)	
*Pain in the evening*	5.9 (1.6)
*Pain interfering with sleep*	3.9 (2.2)

*Note*: Data shown as count (%), mean (SD), or median [range].

Abbreviations: BPI, brief pain inventory; CRPS, complex regional pain syndrome; ISI, Insomnia Severity Index; SD, standard deviation.

The ISI score 1 month after the intervention did not significantly differ from baseline (mean difference in rTMS group −.5, SD 4.6, and in sham group −1.1, SD 3.2; mixed ANOVA main effect of “time” *p* = .43, interaction of “treatment” × “time” *p* = .76). The pain‐related sleep interference during the week before the intervention (median NRS) did not differ from the week after the intervention (mean difference in rTMS group −.17, SD 2.3, and in sham group −1.2, SD 2.0; mixed ANOVA main effect of “time” *p* = .27, interaction of “treatment” × “time” *p* = .40). One month after the intervention, CRPS interference on sleep had decreased in both groups, with no significant difference between the groups (rTMS group mean change −2.0, SD = 2.0; sham mean change −1.6, SD = 2.1; mixed ANOVA main effect of “time” *p* = .003, interaction of “treatment” × “time” *p* = .67).

The pain intensity in NRS after the intervention had decreased in both groups, but there was no significant difference between the rTMS (change in NRS −.44, SD = 1.0) and sham groups (change in NRS −1.6, SD = 1.3, mixed ANOVA main effect of “time” *p* = .003, interaction of “treatment” × “time” *p* = .068).

### 3.2 Oura data

**FIGURE 2:**
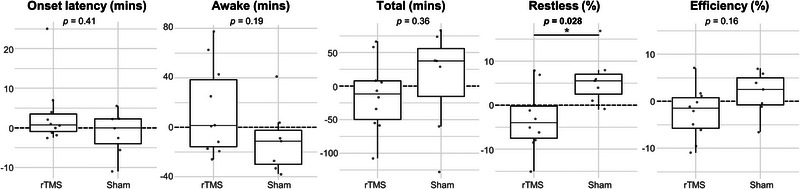
Changes in each Oura parameter after repetitive transcranial magnetic stimulation (rTMS) and sham treatment. Box and whisker plots display the median (thick line), 25%–75% interquartile range (IQR, the boxes), and the smallest and largest values not greater than 1.5*IQR from the hinge (whiskers). “*” denotes *p*‐value less than .05.

Table 2 presents the Oura parameters of 21 patients at baseline. When assessing the effect of the intervention, univariate analysis of changes on individual Oura parameters revealed significant differences between the rTMS and sham groups only for the parameter “restless” (*p* = .028). The effect sizes of rTMS and sham on each Oura parameter are visualized in Figure 2. The change in pain levels correlated inversely with the changes in “restless” (*r* = −.54, *p* = .030), as a greater reduction in pain levels tended to associate with increased restlessness. Other Oura parameters did not correlate significantly with pain reduction.

**TABLE 2:** Baseline Oura data.

Oura variable	
Sleep efficiency (“efficiency”)	88% (6.3%)
Restlessness of sleep time (“restless”)	43% [40%]
Sleep onset latency (“onset_latency”) (min)	7 [12]
Wake after sleep onset (“awake”) (min)	59 (31)
Total sleep time (“total”) (min)	443 (72)
Light sleep (N1 or N2)	55% (12%)
Deep sleep (N3)	12% (10%)
REM sleep	30% (10%)

*Note*: Data shown as group mean (SD) or median [IQR] of the individual pre‐intervention week median values.

Abbreviations: IQR, interquartile range; REM, rapid eye movement.

### 3.3 Oura data: multivariate testing

In the machine learning framework, we evaluated across the seven different models whether LDA would allow separating the two patient groups based on the effects of the rTMS treatment on the sleep data (see Table 3). Out of the seven models, four showed classification accuracies that exceeded the 95% confidence levels. The smallest *p*‐value was observed for the model composed of the variables “onset_latency” and “restless.”

**TABLE 3:** The linear discriminant analysis (LDA)‐based classification results (accuracy, 95% confidence levels and *p*‐values for the significant models) for the seven tested models.

Model variables	Accuracy	95% Confidence level	*p*‐Value
onset_latency, total, restless	59.7788	59.4921	.042
total, restless	60.7976	60.5515	.045
onset_latency, restless	62.4382	61.1129	.027
onset_latency, total	53.9106	59.4726	n.s.
onset_latency	56.7441	61.2021	n.s.
total	56.0112	60.1059	n.s.
restless	62.7894	61.7406	.033

## 4 DISCUSSION

S2‐targeted rTMS influenced sleep in CRPS patients, seen as a reduction of restlessness as measured by the Oura ring. Furthermore, multivariate testing using machine learning‐based classification showed that four out of the seven tested models led to classification accuracies exceeding 95% confidence levels. Thus, monitoring changes across multiple Oura variables allowed separating the rTMS and sham groups from each other.

Chronic pain with impaired quality of sleep is a well‐known problem, and they interact in a bidirectional manner (Bjurstrom & Irwin, 2016). Individuals with chronic pain are, for example, 18 times more likely to have insomnia than those without chronic pain (Tang et al., 2007). On the other hand, sleep disturbances may affect key processes in chronic pain maintenance including microglia activation and endogenous pain inhibition (Finan et al., 2013; Huang et al., 2014). Disrupted sleep continuity is a frequent finding in patients with chronic pain, and PSG studies have revealed increased light sleep and decreased slow‐wave sleep. However, these changes in sleep architecture have been inconsistent (Bjurstrom & Irwin, 2016).

Data about sleep among CRPS patients is scarce, consisting mostly of subjective sleep measurements. Galer et al. (2000) reported that the majority of CRPS patients suffered from sleep disturbance. Their estimation of sleep interference (BPI score 6.1) was of similar magnitude as in our patient cohort (Table 1). Objective sleep measurements in CRPS patients, however, are lacking. Therefore, our baseline Oura data (Table 2) are a valuable data set by itself, providing objective insight into sleep in patients with CRPS. With consumer‐grade wearable devices becoming increasingly popular (Miller et al., 2022), more reports on Oura results (and other consumer wearables) in various conditions are likely to emerge and comparison, for example, among different pain conditions will become feasible.

The effect of rTMS on sleep is complex and depends on the rTMS target area and the chosen stimulation protocol, among others. For example, in PI, rTMS targeted to the dorsolateral prefrontal cortex might inhibit the over‐excited state associated with PI, and this could lead to improved sleep (Nardone et al., 2020; Oroz et al., 2021). The neurophysiologic effects of S2‐targeted rTMS are poorly studied. rTMS in general can modulate the level of several neurotransmitters (e.g., Malik et al., 2018; Michael et al., 2003; Poh et al., 2019), and some evidence suggests that S2‐targeted rTMS increases excitatory neurotransmitter levels (Fregni et al., 2011). In addition, the functional connectivity of the rTMS target area might also be important for the rTMS effect, and the analgesic effect of S2‐targeted rTMS has been suggested to be attributed to the rich interconnections between S2 and insula (Fregni et al., 2011; Lindholm et al., 2015).

S2‐targeted rTMS was significantly more effective than sham in decreasing restlessness during sleep. Univariate analysis did not reveal other significant changes in the Oura‐measured parameters, but our machine learning‐assisted multivariate analysis revealed that including multiple parameters in the classification improved results. The model utilizing parameters “restless” and “onset_latency” yielded classification accuracy with the lowest *p*‐value, and two other multivariate models were statistically significant as well. Thus, we interpret that even though a univariate model could in this study reveal the difference in the effect on sleep between the rTMS and sham group, inclusion of multiple sleep‐related parameters can add relevant information and improve the analysis of rTMS effects on sleep. The effect of rTMS on sleep was not captured in the ISI questionnaire or in the pain–sleep diary. This highlights the importance of objective sleep measurements, to better understand the neurophysiological effects of noninvasive neuromodulative treatments.

In this study sample, the analgesic effect of S2‐targeted rTMS did not differ from sham. Pain reduction, surprisingly, associated with an increase in sleep restlessness, but other sleep‐related parameters did not correlate with the pain reduction. Thus, the beneficial effect of S2‐targeted rTMS on sleep does not seem to be secondary to the possible analgesic effect of the intervention. A recent fMRI‐EEG study suggested that insula, among other brain areas, is part of a network that affects arousability during sleep (Kokkinos et al., 2019). The connections of S2 with insula could thus provide an explanation to why S2‐targeted rTMS could independently decrease restlessness during sleep. We targeted only the right S2 with rTMS because some evidence suggests a lateralization of the pain matrix and an analgesic effect of S2‐stimulation on the right side (Fregni et al., 2005; Hsieh et al., 1995; Symonds et al., 2006), but the effect of left S2 stimulation on sleep remains unknown.

A number of studies have revealed neurophysiological abnormalities at the cortical level in CRPS (Zangrandi et al., 2021). Several primary sleep disorders also share some of these aspects with CRPS (Lanza et al., 2015) and, for example, RLS and CRPS both associate with reduced intracortical inhibition of the sensorimotor cortex. We did not evaluate the presence of RLS symptoms in our patients, but a natural future progression would be to assess whether S2‐targeted rTMS can improve sleep in primary sleep disorders, or RLS specifically, considering its association with the sensorimotor system.

### 4.1 Methodological considerations

The rTMS effect on sleep was assessed with two subjective items and four objective Oura variables. None of these were predefined as a primary outcome measure, and thus concurrent analysis of several parameters increases the risk of false‐positive results. The sample size for this study was defined by the power analysis of the multicenter study, utilizing different outcome variables than in this study.

As the most significant LDA‐based model utilized multiple variables (“restless” and “onset_latency”), the findings suggest that it is beneficial to consider simultaneously multiple sleep measures when examining the effects of rTMS treatment on sleep. However, as the variable “restless” that yielded significant results in the univariate analysis was included in all the LDA‐based models with significant findings, the possible benefits of multivariate analyses in examining the effects of rTMS treatment on sleep need to be verified in a larger patient cohort.

The small patient cohort limited the number of Oura variables that could be considered in the analyses. The Oura ring provides 13 original measures that could have been used in the multivariate analyses. As our sample consisted of a total of 17 patients, we restricted the testing to the four most likely candidates based on previous studies. However, a recent validation study suggests that several consumer wearables, including the Oura ring, provide reasonable estimations of sleep stage classification as well (Miller et al., 2022), and this could perhaps be included in the analysis models in the future. In a larger cohort, all possible measures and their combinations could be considered, allowing the identification of the most relevant and robust measures to follow the effects of rTMS‐based intervention on sleep.

Another limiting factor was the small amount of data from individual patients. On average, Oura data were collected during about five and four nights in the pre‐ and posttreatment phases, respectively. However, in several patients, data were available only from three nights either in the pre‐ or posttreatment phase. Thus, the LDA‐based analyses were conducted using data from individual nights only in both the training and testing phase. Accordingly, the classification analyses were likely considerably affected by random variability in the sleep patterns across individual nights. The collection of Oura data for longer periods both in the pre‐ and‐post treatment phases would allow, for example, averaging of data from individual nights, leading to more stable estimates of the Oura variables and more robust dissociation of the patient groups as well as more robust identification of the key sleep measures.

As no general normative values for Oura variables exist, we cannot determine if the sleep‐related values in CRPS patients were abnormal as compared with people without pain. Additionally, as there was no significant improvement in self‐reported sleep after the intervention, the clinical relevance of the S2‐targeted rTMS effect on sleep remains to be studied in more detail.

## 5 CONCLUSIONS

S2‐targeted rTMS might be beneficial in improving sleep in CRPS patients. A machine learning–based analysis across multiple Oura variables could be used to dissociate the rTMS and sham groups from each other. Thus, considering multiple sleep‐related parameters is worthwhile to increase knowledge of the neurophysiological effects of noninvasive neuromodulative treatments. Future studies with larger samples and other rTMS targets are required to confirm these results and to better understand the interplay of sleep and rTMS effects in CRPS.

## AUTHOR CONTRIBUTIONS

Jukka Vanhanen, Hanna Harno, and Eija Kalso conceived the study. Hanna Harno recruited the patients, Jukka Vanhanen and Hanna Harno collected the data. Jukka Vanhanen, Jan Kujala, Mia Liljeström, and Jussi Virkkala analyzed the data. Jukka Vanhanen drafted the manuscript. All authors participated in data interpretation, revised the article critically, and approved the final version of the article.

## CONFLICT OF INTEREST STATEMENT

The authors have no conflicts of interest to declare.

## FUNDING INFORMATION

The state funding for university‐level health research [TYH2016222, TYH2021‐215], the Biomedicum Helsinki Foundation [20220071], the Signe and Ane Gyllenberg Foundation [5672], and the Maud Kuistila Memorial Foundation [2022‐0031B]

## CLINICAL TRIAL REGISTRATION

The study was registered in the ClinicalTrials.gov (NCT04439669).

### PEER REVIEW

The peer review history for this article is available at https://publons.com/publon/10.1002/brb3.3252.

## Data Availability

Ethical restrictions imposed by the hospital's research ethics committee prevent the authors from making raw data publicly available without restrictions. However, the relevant summary tables of the data are available from the authors upon reasonable request and with permission of the hospital's research ethics committee, for researchers aiming to reproduce the results.
